# Supplementary material for: Cryo-ET and MD simulations reveal that dynein-2 is tuned for binding to the A-tubule of the ciliary doublet
Source: EMBO J. 2025 Nov 26;44(24):7677–701. doi: 10.1038/s44318-025-00648-1 (PMC12706001; doi:10.1038/s44318-025-00648-1)
Supplement: Supplementary file 2 — Movie EV1 [file 44318_2025_648_MOESM2_ESM.zip › Movie EV1.docx]

**Movie EV1.**

Aligned tilt-series images of GST-Dyn2 decorated doublets collected with VPP.
