# Supplementary figures and images for: Cryo-ET and MD simulations reveal that dynein-2 is tuned for binding to the A-tubule of the ciliary doublet
Source: EMBO J. 2025 Nov 26;44(24):7677–701. doi: 10.1038/s44318-025-00648-1 (PMC12706001; doi:10.1038/s44318-025-00648-1)

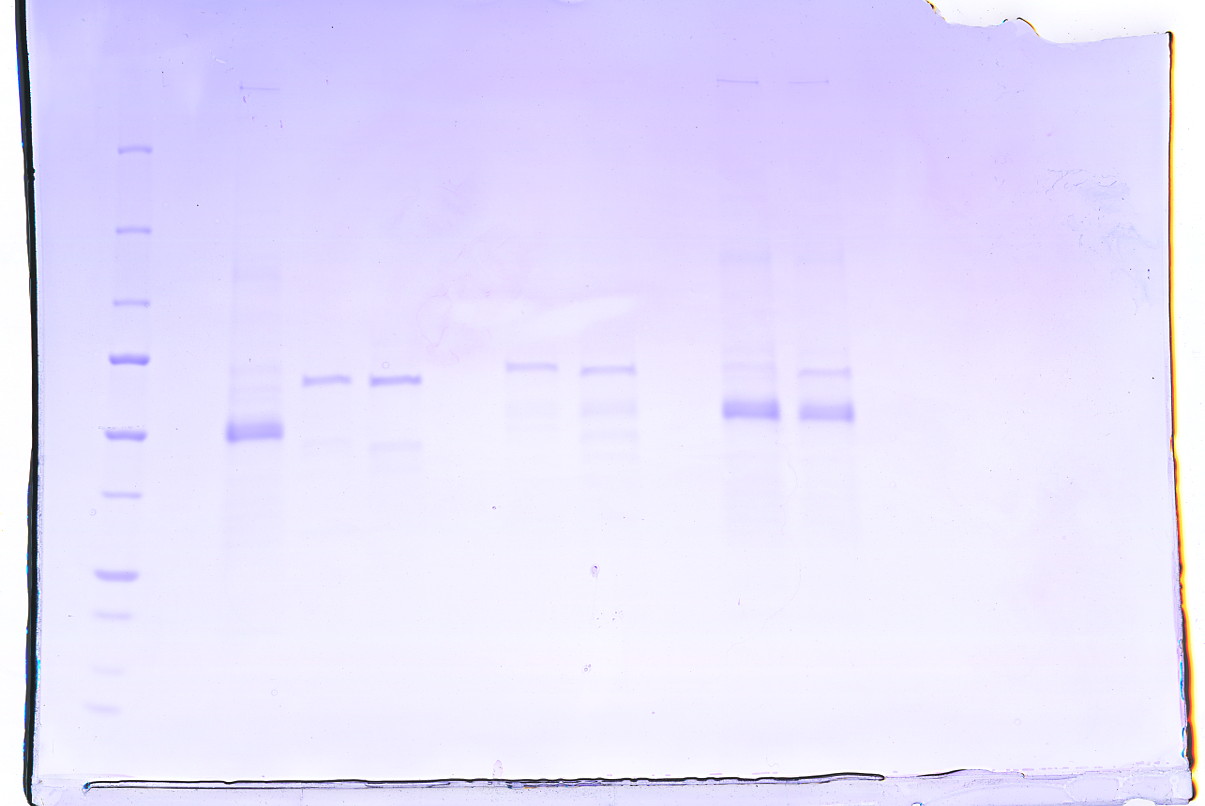

Supplement: Supplementary file 4 — Source data Fig. 2 [file 44318_2025_648_MOESM4_ESM.zip › Figure 2/2E/gel_image.tif]

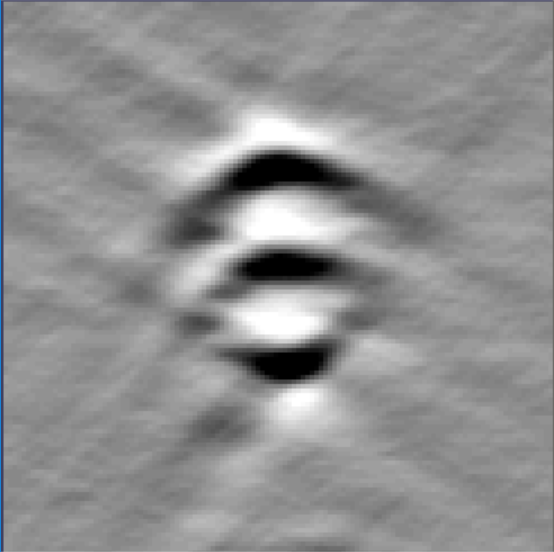

Supplement: Supplementary file 5 — Source data Fig. 3 [file 44318_2025_648_MOESM5_ESM.zip › Figure 3/3E/Doublet_3.PNG]

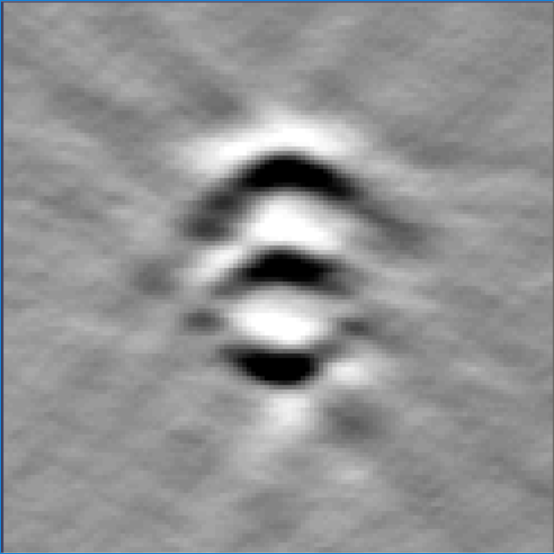

Supplement: Supplementary file 5 — Source data Fig. 3 [file 44318_2025_648_MOESM5_ESM.zip › Figure 3/3E/Doublet_2.png]

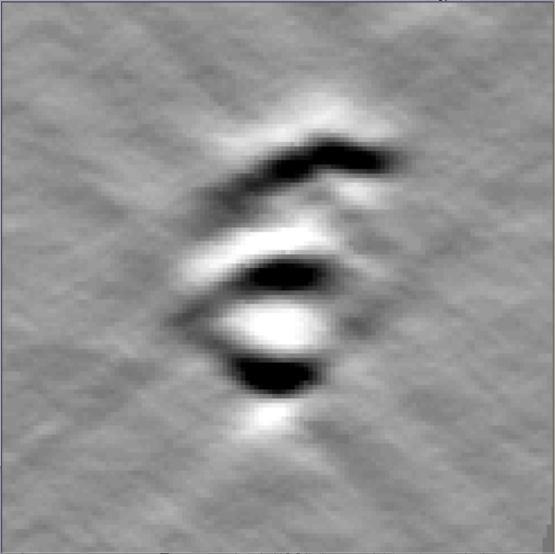

Supplement: Supplementary file 5 — Source data Fig. 3 [file 44318_2025_648_MOESM5_ESM.zip › Figure 3/3E/Doublet_1.PNG]

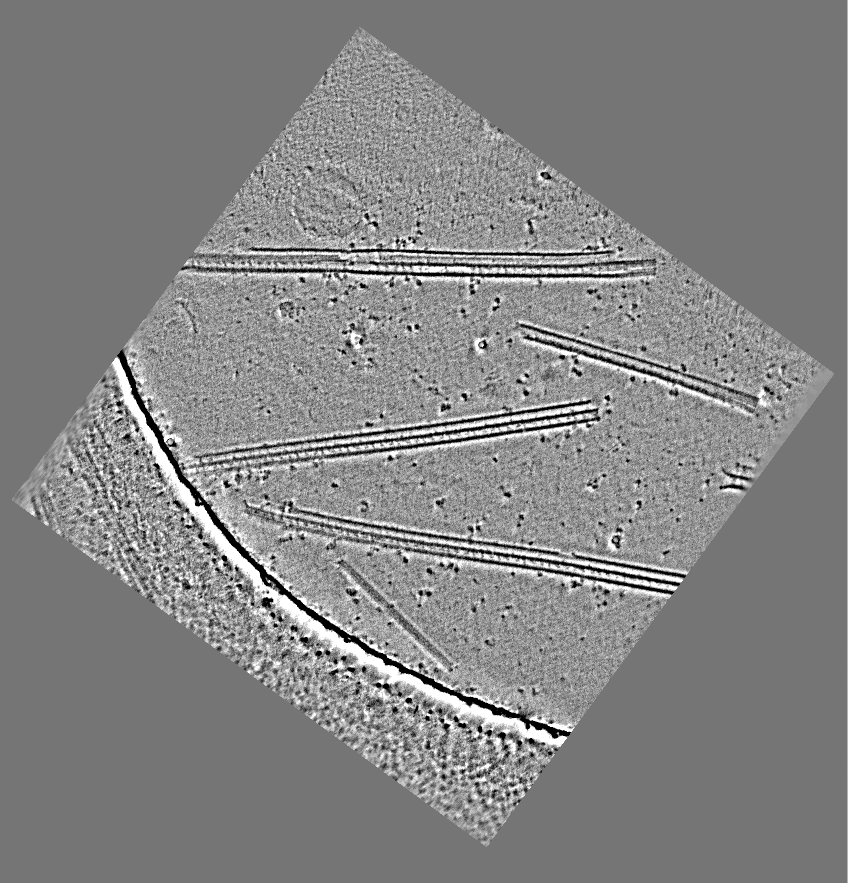

Supplement: Supplementary file 5 — Source data Fig. 3 [file 44318_2025_648_MOESM5_ESM.zip › Figure 3/3E/General_image.png]

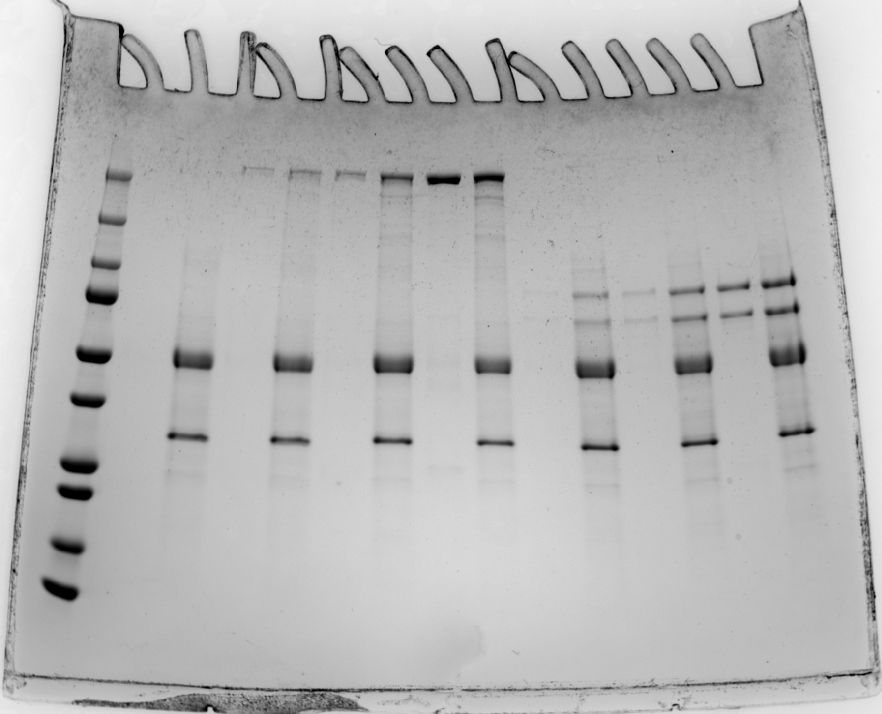

Supplement: Supplementary file 5 — Source data Fig. 3 [file 44318_2025_648_MOESM5_ESM.zip › Figure 3/3C/gel_image.tif]

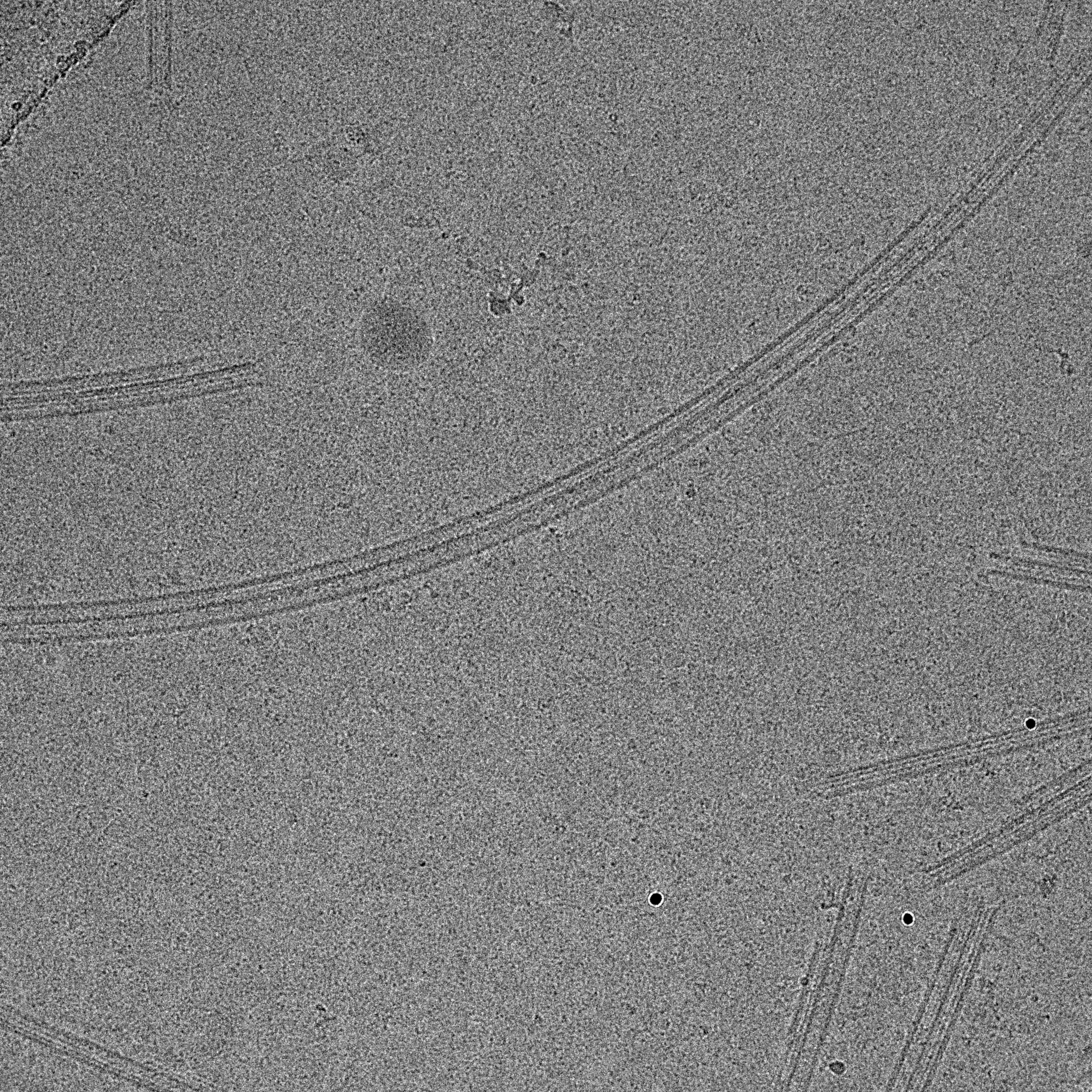

Supplement: Supplementary file 5 — Source data Fig. 3 [file 44318_2025_648_MOESM5_ESM.zip › Figure 3/3D/EM_image.tiff]

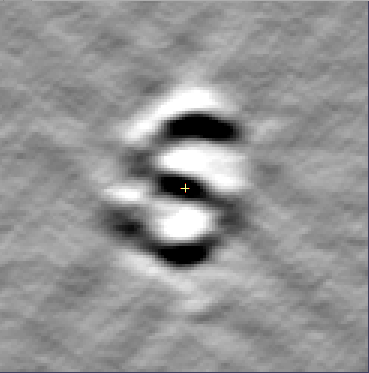

Supplement: Supplementary file 5 — Source data Fig. 3 [file 44318_2025_648_MOESM5_ESM.zip › Figure 3/3F/Doublet3_cross-section.png]

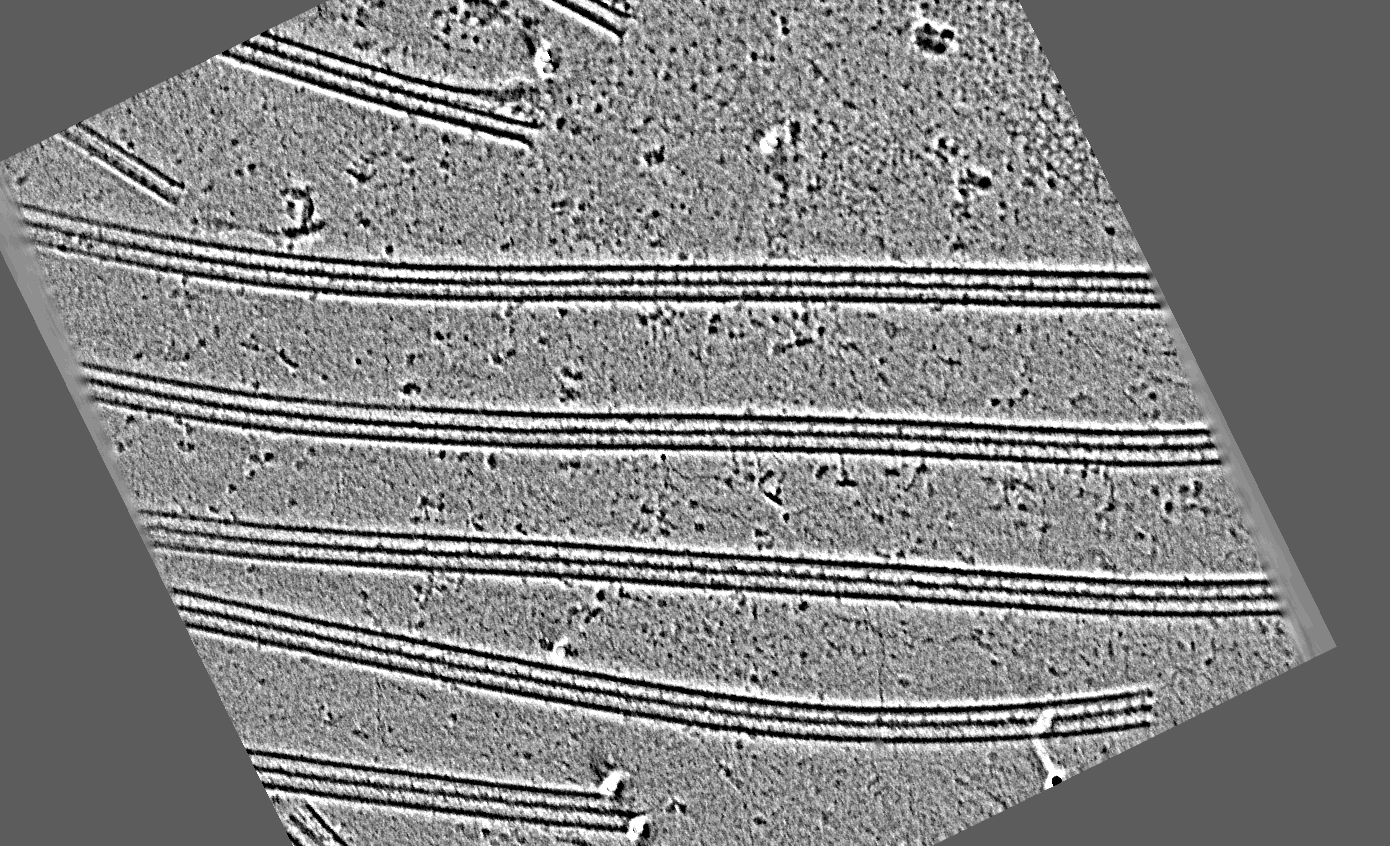

Supplement: Supplementary file 5 — Source data Fig. 3 [file 44318_2025_648_MOESM5_ESM.zip › Figure 3/3F/Doublet1_longitudinal.png]

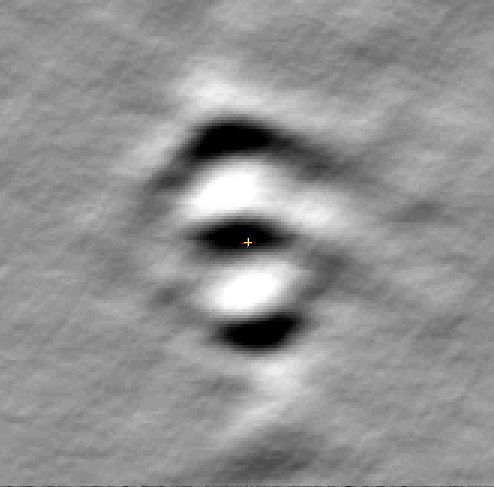

Supplement: Supplementary file 5 — Source data Fig. 3 [file 44318_2025_648_MOESM5_ESM.zip › Figure 3/3F/Doublet2_cross-section.png]

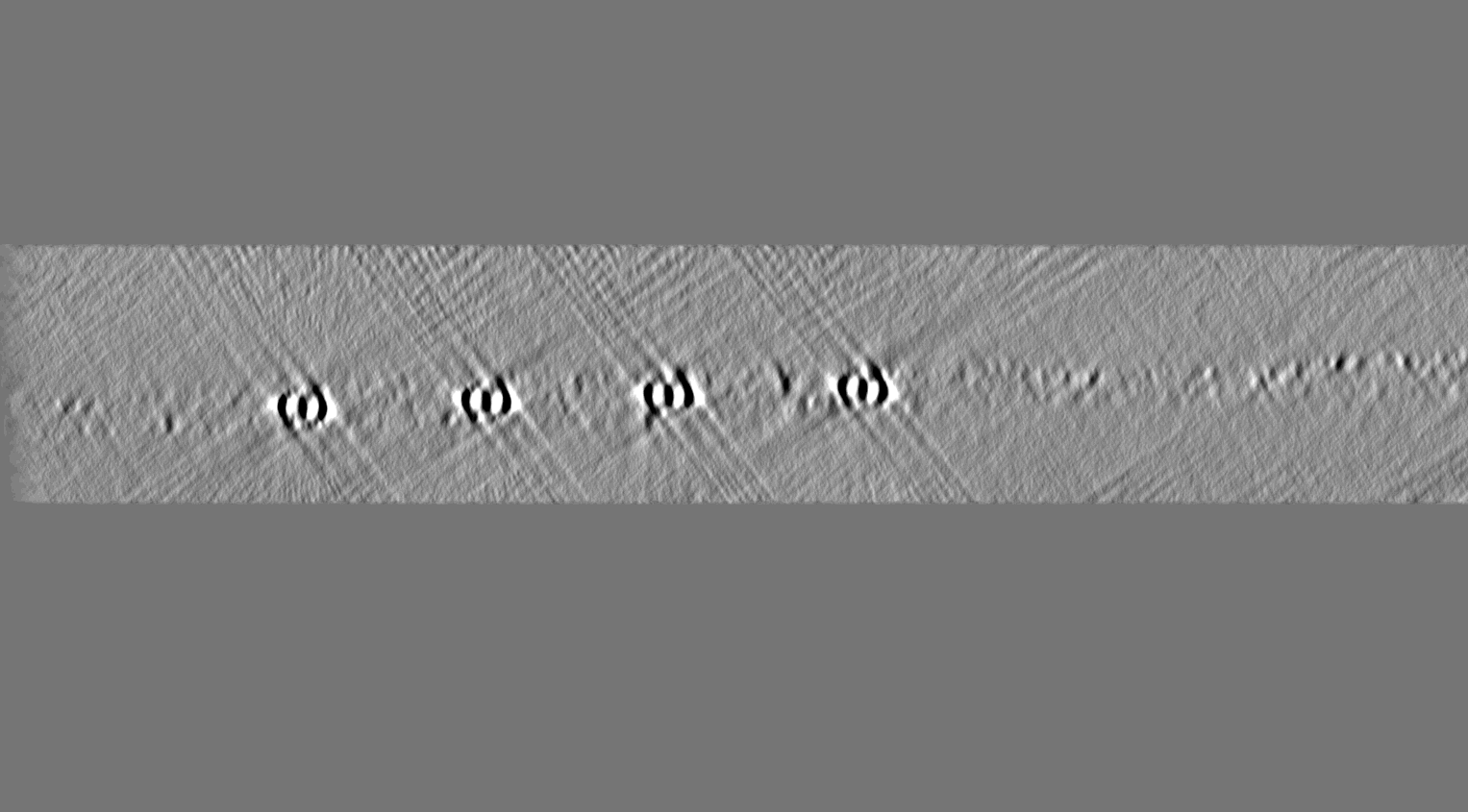

Supplement: Supplementary file 5 — Source data Fig. 3 [file 44318_2025_648_MOESM5_ESM.zip › Figure 3/3F/Doublet1_cross-section.png]

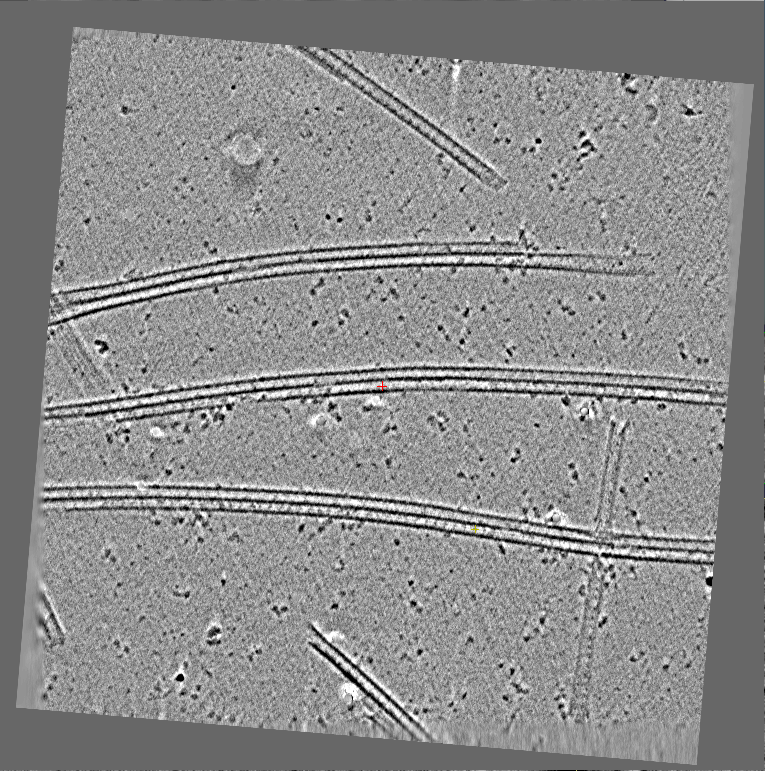

Supplement: Supplementary file 5 — Source data Fig. 3 [file 44318_2025_648_MOESM5_ESM.zip › Figure 3/3F/Doublet3_longitudinal.png]

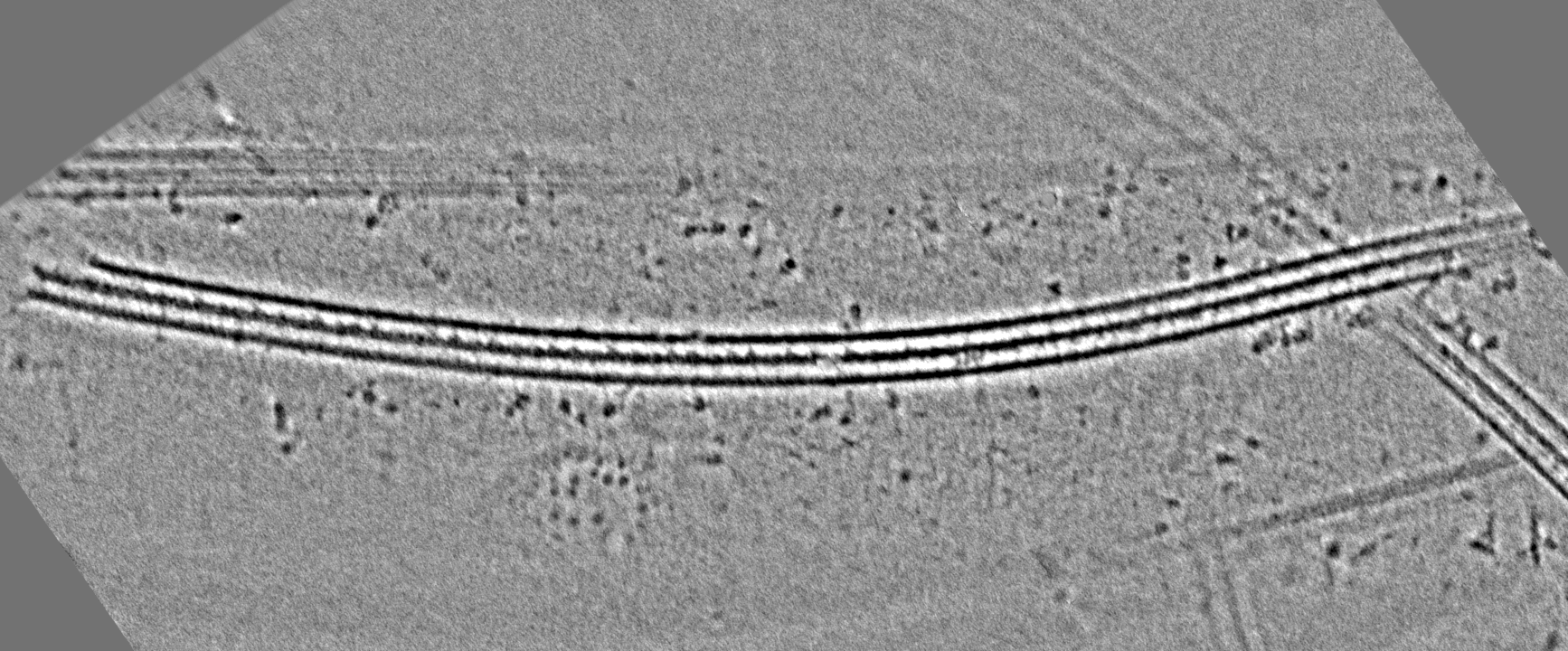

Supplement: Supplementary file 5 — Source data Fig. 3 [file 44318_2025_648_MOESM5_ESM.zip › Figure 3/3F/Doublet2_longitudinal.png]

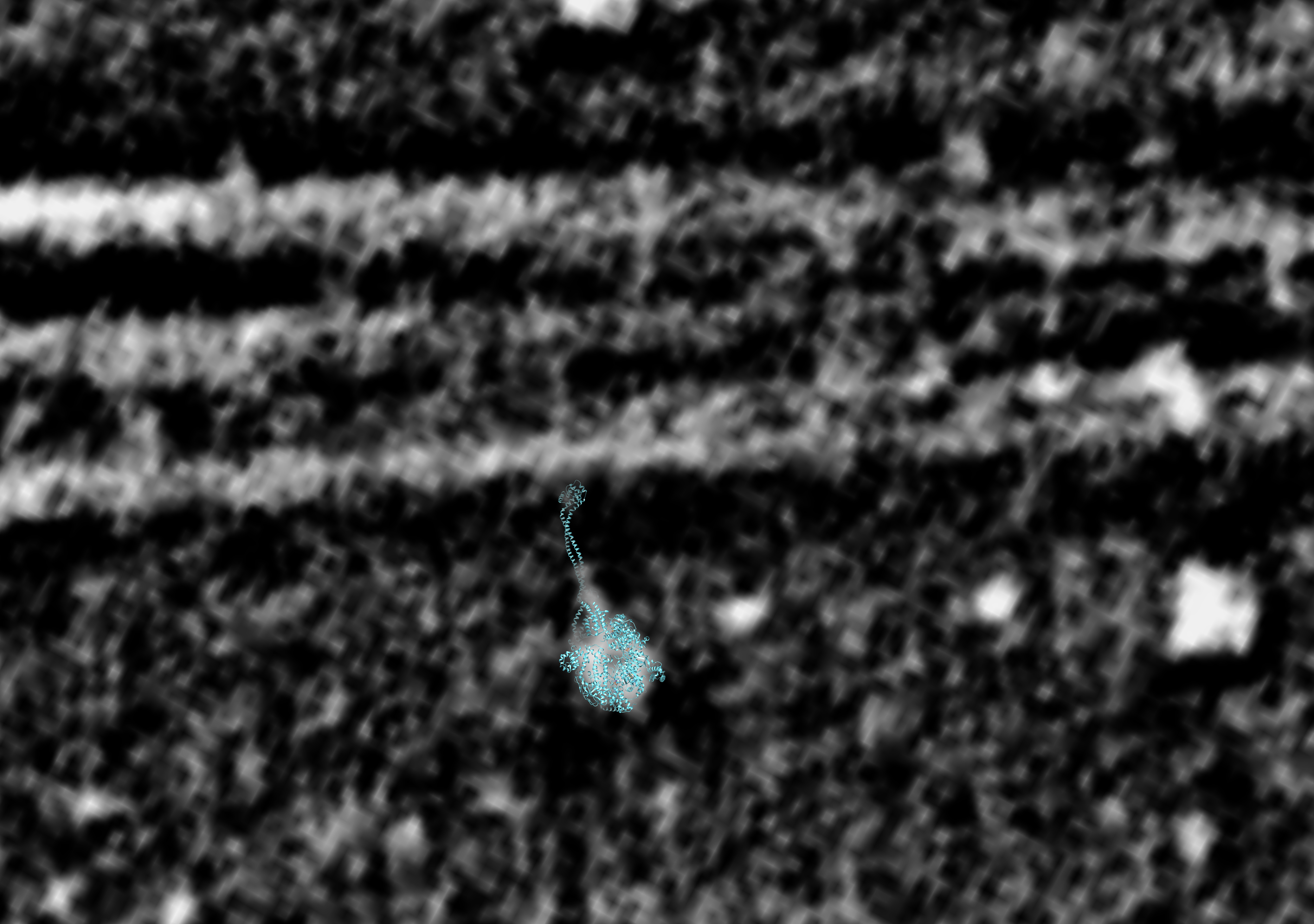

Supplement: Supplementary file 5 — Source data Fig. 3 [file 44318_2025_648_MOESM5_ESM.zip › Figure 3/3G/Image_2.png]

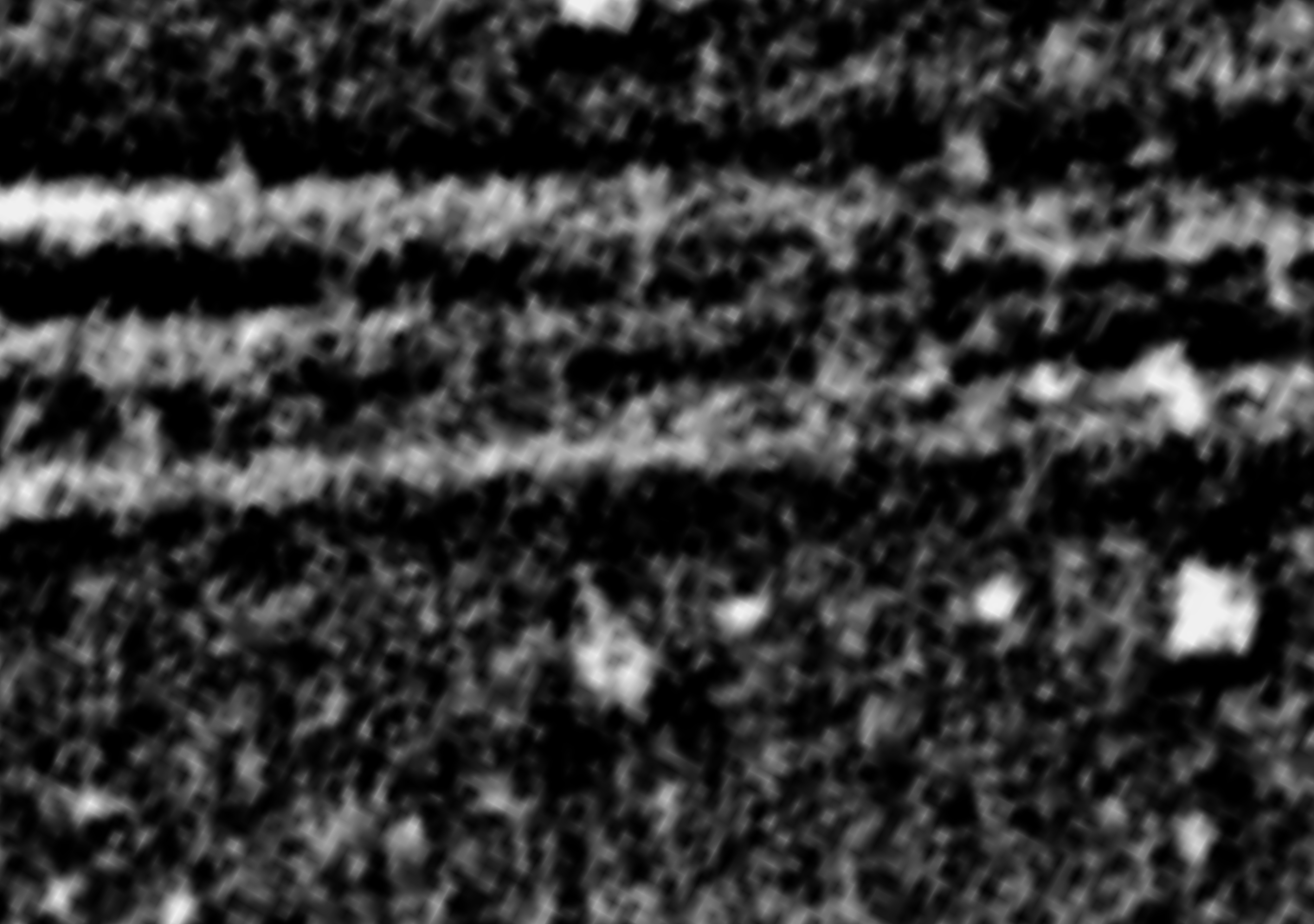

Supplement: Supplementary file 5 — Source data Fig. 3 [file 44318_2025_648_MOESM5_ESM.zip › Figure 3/3G/Image_1.png]

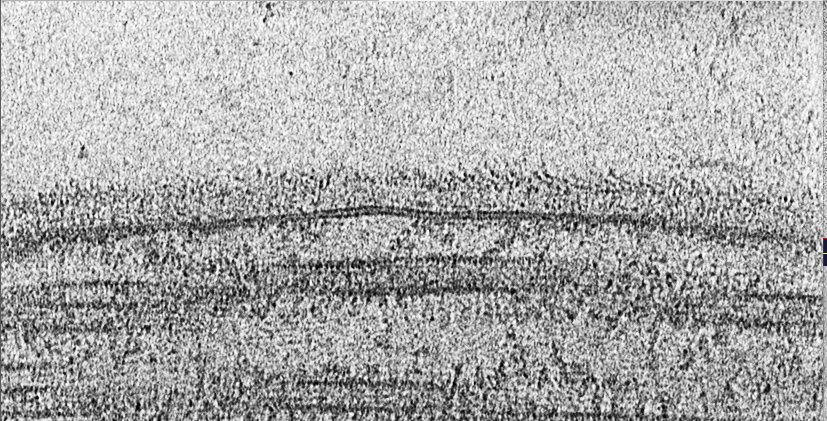

Supplement: Supplementary file 6 — Source data Fig. 4 [file 44318_2025_648_MOESM6_ESM.zip › Figure 4/4C/Image.png]

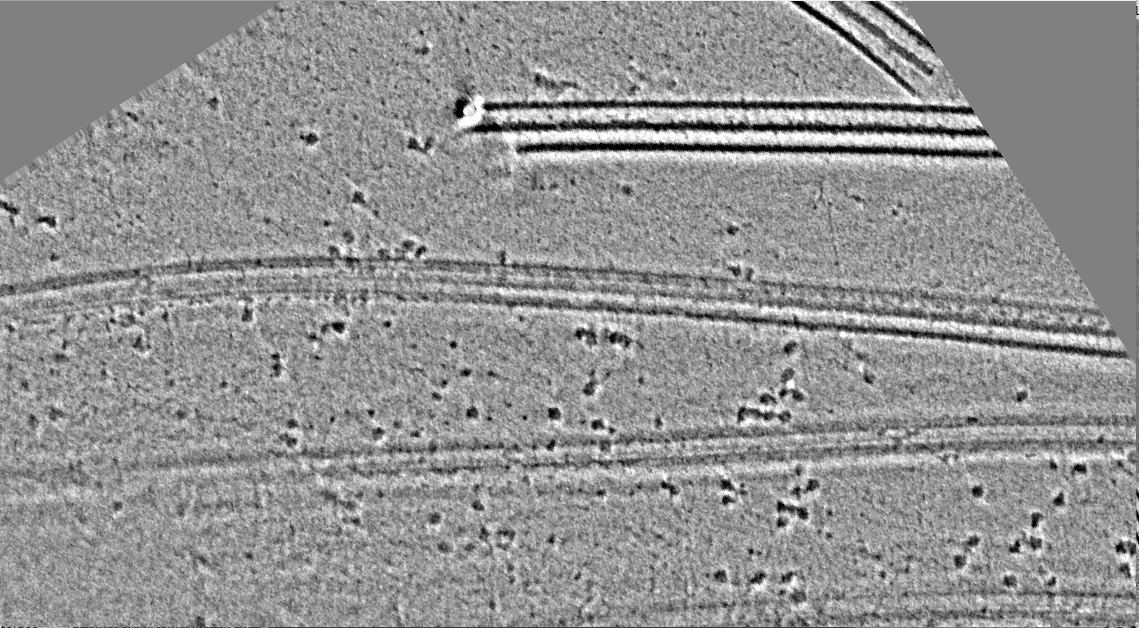

Supplement: Supplementary file 6 — Source data Fig. 4 [file 44318_2025_648_MOESM6_ESM.zip › Figure 4/4A/Image_2.png]

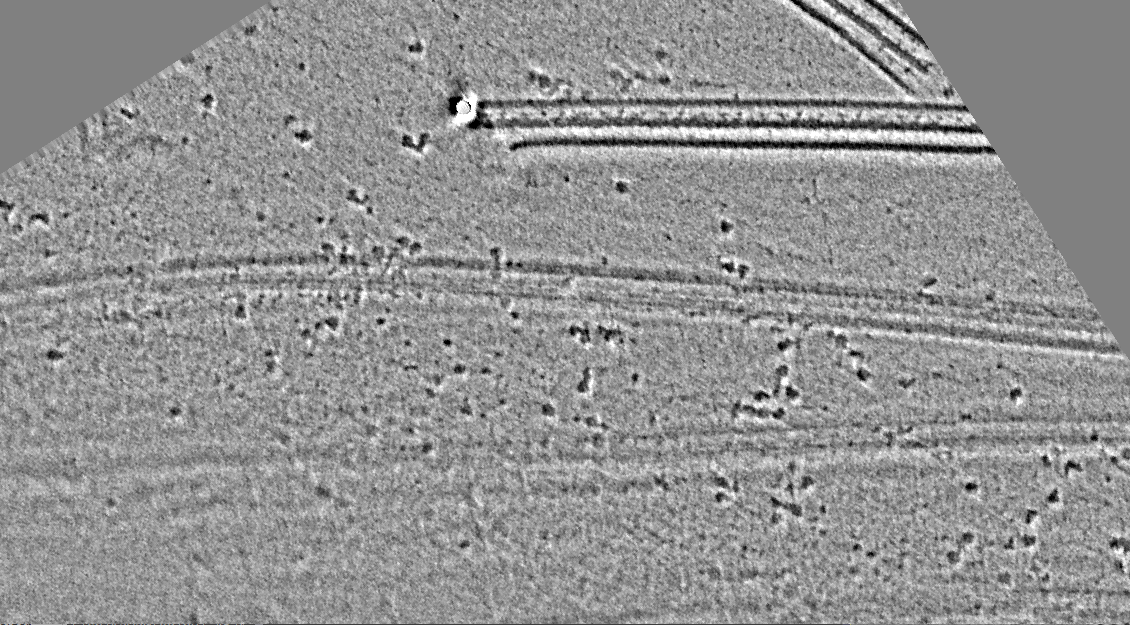

Supplement: Supplementary file 6 — Source data Fig. 4 [file 44318_2025_648_MOESM6_ESM.zip › Figure 4/4A/Image_3.png]

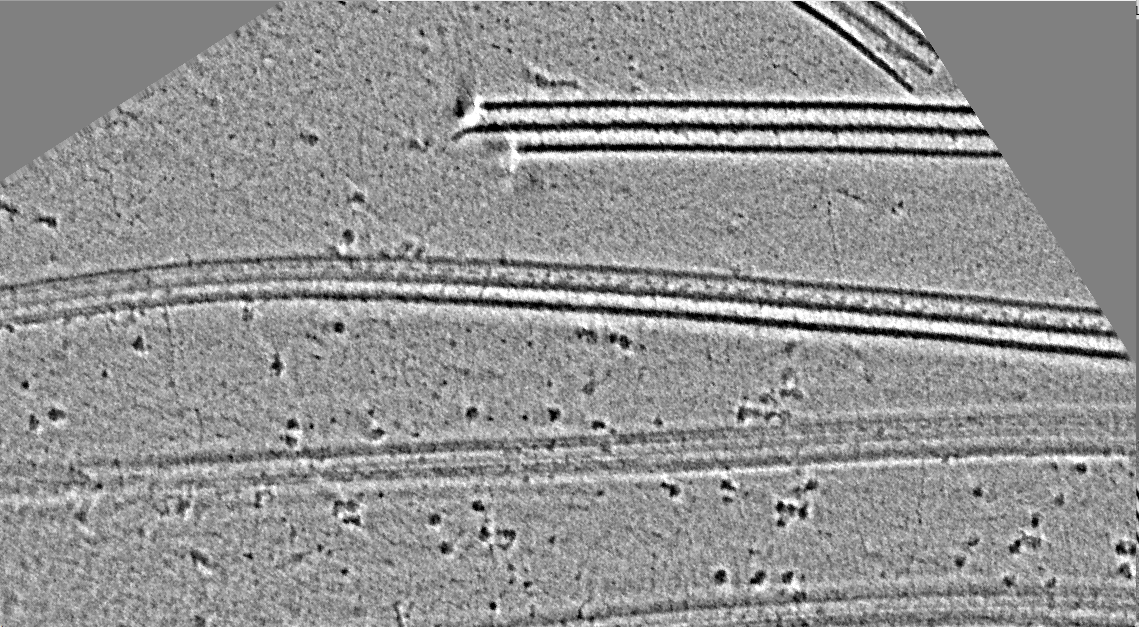

Supplement: Supplementary file 6 — Source data Fig. 4 [file 44318_2025_648_MOESM6_ESM.zip › Figure 4/4A/Image_1.png]

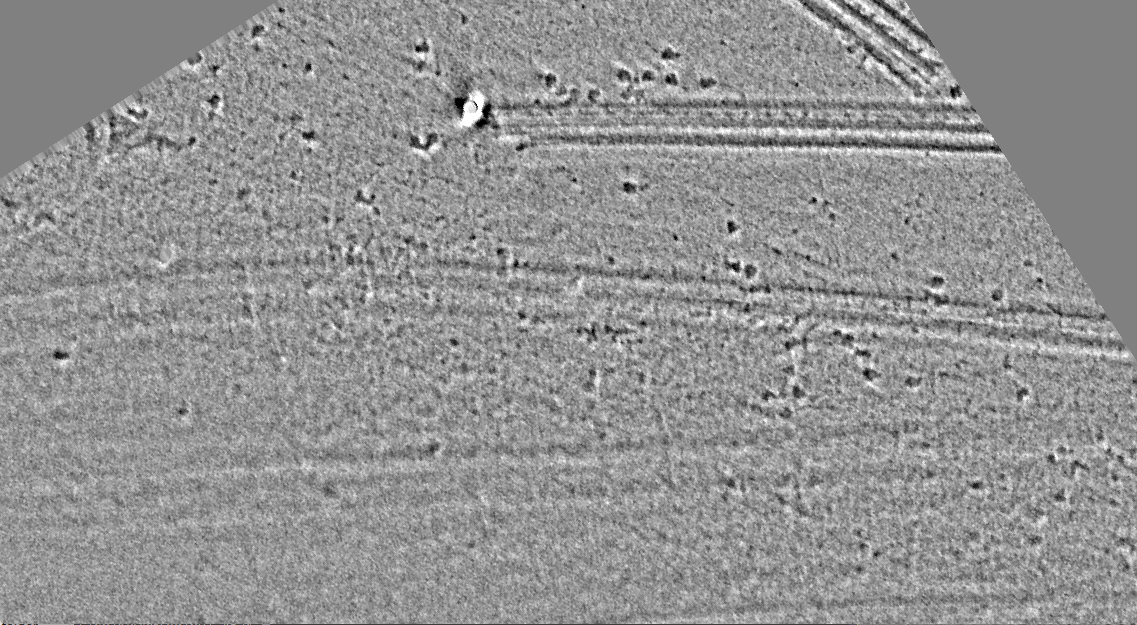

Supplement: Supplementary file 6 — Source data Fig. 4 [file 44318_2025_648_MOESM6_ESM.zip › Figure 4/4A/Image_4.png]

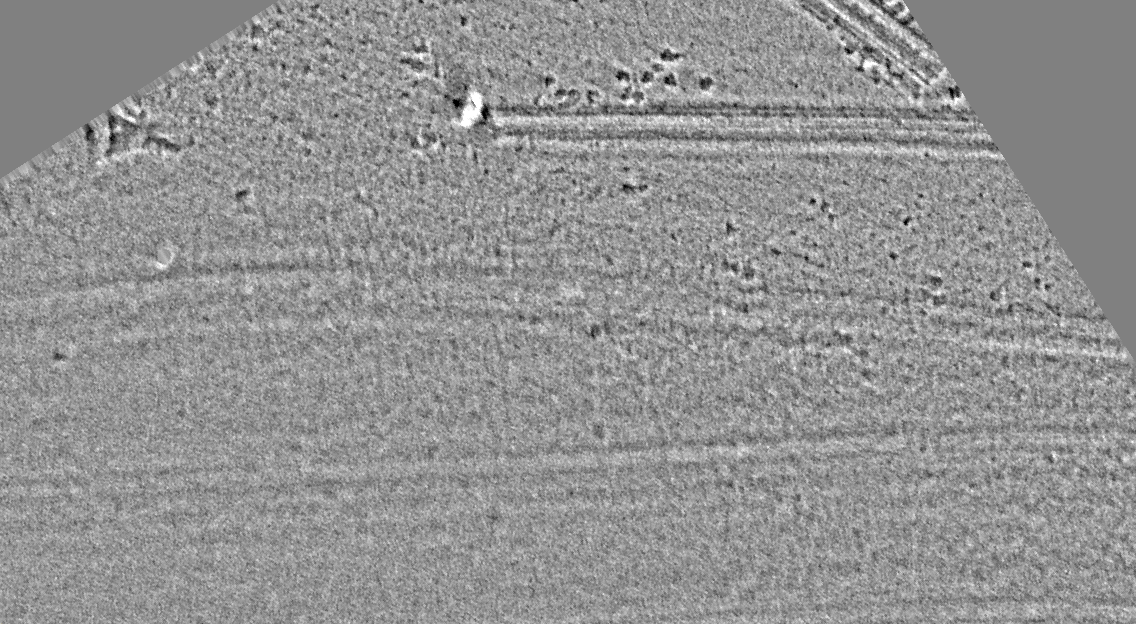

Supplement: Supplementary file 6 — Source data Fig. 4 [file 44318_2025_648_MOESM6_ESM.zip › Figure 4/4A/Image_5.png]
