# Supplementary material for: Cryo-ET and MD simulations reveal that dynein-2 is tuned for binding to the A-tubule of the ciliary doublet
Source: EMBO J. 2025 Nov 26;44(24):7677–701. doi: 10.1038/s44318-025-00648-1 (PMC12706001; doi:10.1038/s44318-025-00648-1)
Supplement: Supplementary file 8 — Expanded View Figures [file 44318_2025_648_MOESM8_ESM.pdf]

Expanded View Figures

Figure EV1. Sequence alignment of MTBD regions from dynein-2 and dynein-1 HCs.

Amino acid sequence of the MTBD region of human dynein-2 HC (NCBI Reference Sequence: NP_001368.2), *Mus musculus* dynein-2 HC (NCBI Reference Sequence: NP_084127.2), *Danio rerio* dynein-2 HC (NCBI Reference Sequence: NP_001410228), *Xenopus laevis* dynein-2 HC (NCBI Reference Sequence: XP_041438615), *Drosophila melanogaster* dynein-2 HC (NCBI Reference Sequence: NP_001036369), *Caenorhabditis elegans* dynein-2 HC (NCBI Reference Sequence: NP_492221.2), *Chlamydomonas reinhardtii* dynein-1b HC (NCBI Reference Sequence: XP_001696428.1), human dynein-1 HC (NCBI Reference Sequence: NP_001367.2), *Mus musculus* dynein-1 HC (NCBI Reference Sequence: NP_084514.2), *Xenopus laevis* dynein-1 HC (NCBI Reference Sequence: XP_018086051.1), *Danio rerio* dynein-1 HC (NCBI Reference Sequence: NP_001036210.1), *Drosophila melanogaster* dynein-1 HC (NCBI Reference Sequence: NP_001261430.1), *Caenorhabditis elegans* dynein-1 HC (NCBI Reference Sequence: NP_491363.1) were aligned using Clustal W (Thompson et al, 1994) and figure was prepared using ESPript 3.0 (Robert and Gouet, 2014). Note that *Chlamydomonas reinhardtii* dynein-1b is IFT dynein equivalent to dynein-2. Cyan highlights show the amino acid residues conserved only in dynein-2 and yellow highlights indicate the amino acid residues conserved only in dynein-1. White arrowheads indicate the residues conserved either in dynein-2 or dynein-1.

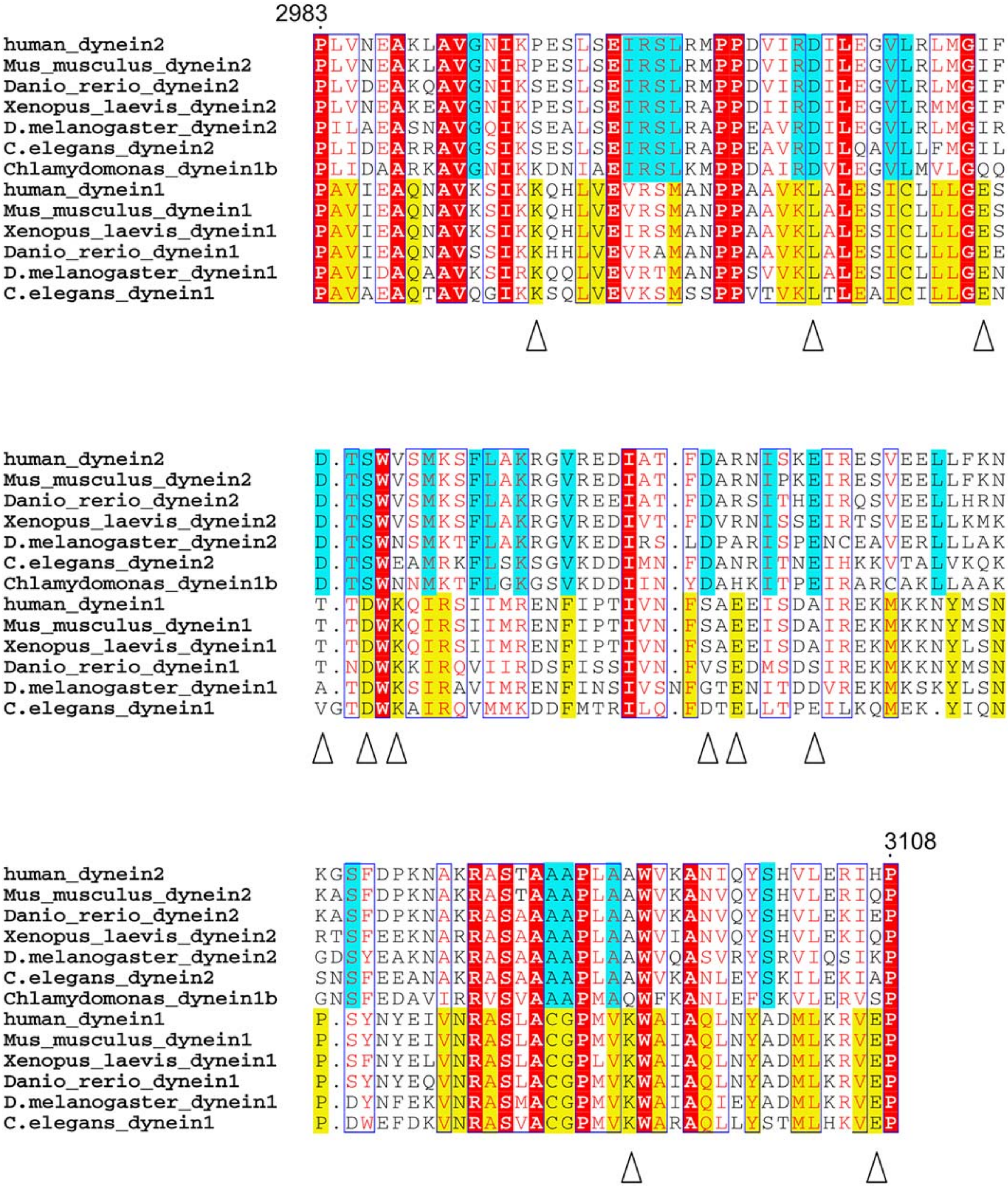
2983
human_dynein2 PLVNEAKLAVGNIKPESLSEIRSLRMPPDVIRDILEGVLRLMGIF
Mus_musculus_dynein2 PLVNEAKLAVGNIRPESLSEIRSLRMPPDVIRDILEGVLRLMGIF
Danio_rerio_dynein2 PLVDEAKQAVGNIKSESLSEIRSLRMPPDVIRDILEGVLRLMGIF
Xenopus_laevis_dynein2 PLVNEAKEAVGNIKPESLSEIRSLRAPPDIIRDILEGVLRMMGIF
D.melanogaster_dynein2 PILAEASNAVGQIKSEALSEIRSLRAPPEAVRDILEGVLRLMGIR
C.elegans_dynein2 PLIDEARRAVGSIKSESLSEIRSLRAPPEAVRDILQAVLLFMGIL
Chlamydomonas_dynein1b PLIDAARKAVGNIKKDNIAEIRSLKMPPDAIRDVLEGVLMVLGQQ
human_dynein1 PAVIEAQNAVKSIKKQHLVEVRSMANPPAAVKLALESICLLLGES
Mus_musculus_dynein1 PAVIEAQNAVKSIKKQHLVEVRSMANPPAAVKLALESICLLLGES
Xenopus_laevis_dynein1 PAVIEAQNAVKSIKKQHLVEIRSMANPPAAVKLALESICLLLGES
Danio_rerio_dynein1 PAVIEAQNAVSSIKKHHLVEVRAMANPPAAVKLALESICLLLGEE
D.melanogaster_dynein1 PAVIDAQAAVKSIRKQQLVEVRTMANPPSVVKLALESICLLLGEN
C.elegans_dynein1 PAVAEAQTAVQGIKKSQLVEVKSMSSPPVTVKLTLEAICILLGEN
human_dynein2 D.TSWVSMKSFLAKRGVREDIAT.FDARNISKEIRESVEELLFKN
Mus_musculus_dynein2 D.TSWVSMKSFLAKRGVREDIAT.FDARNIPKEIRESVEELLFKN
Danio_rerio_dynein2 D.TSWVSMKSFLAKRGVREEIAT.FDARSITHEIRQSVEELLHRN
Xenopus_laevis_dynein2 D.TSWVSMKSFLAKRGVREDIVT.FDVRNISSEIRTSVEELLKMK
D.melanogaster_dynein2 D.TSWNSMKTFLAKRGVKEDIRS.LDPARISPENCEAVERLLLAK
C.elegans_dynein2 D.TSWEAMRKFLSKSGVKDDIMN.FDANRITNEIHKKVTALVKQK
Chlamydomonas_dynein1b D.TSWNNMKTFLGKGSVKDDIIN.YDAHKITPEIRARCAKLLAAK
human_dynein1 T.TDWKQIRSIIMRENFIPTIVN.FSAEEISDAIREKMKKNYMSN
Mus_musculus_dynein1 T.TDWKQIRSIIMRENFIPTIVN.FSAEEISDAIREKMKKNYMSN
Xenopus_laevis_dynein1 T.TDWKQIRSIIMRENFIPTIVN.FSAEEISDAIREKMKKNYLSN
Danio_rerio_dynein1 T.NDWKKIRQVIIRDSFISSIVN.FVSEDMSDSIREKMKKNYMSN
D.melanogaster_dynein1 A.TDWKSIRAVIMRENFINSIVSNFGTENITDDVREKMKSKYLSN
C.elegans_dynein1 VGTDWKAIRQVMMKDDFMTRILQ.FDTELLTPEILKQMEK.YIQN
3108
human_dynein2 KGSFDPKNAKRASTAAAPLAAWVKANIQYSHVLERIHP
Mus_musculus_dynein2 KASFDPKNAKRASTAAAPLAAWVKANVQYSHVLERIQP
Danio_rerio_dynein2 KASFDPKNAKRASAAAAPLAAWVKANVQYSHVLEKIEP
Xenopus_laevis_dynein2 RTSFEEKNARRASAAAAPLAAWVIANVQYSHVLEKIQP
D.melanogaster_dynein2 GDSYEAKNAKRASAAAAPLAAWVQASVRYSRVIQSIKP
C.elegans_dynein2 SNSFEEANAKRASAAAAPLAAWVKANLEYSKILEKIAP
Chlamydomonas_dynein1b GNSFEDAVIRRVSVAAAPMAQWFKANLEFSKVLERVSP
human_dynein1 P.SYNYEIVNRASLACGPMVKWAIAQLNYADMLKRVEP
Mus_musculus_dynein1 P.SYNYEIVNRASLACGPMVKWAIAQLNYADMLKRVEP
Xenopus_laevis_dynein1 P.SFNYELVNRASLACGPLVKWAIAQLNYADMLKRVEP
Danio_rerio_dynein1 P.SYNYEQVNRASLACGPMVKWAIAQLNYADMLKRVEP
D.melanogaster_dynein1 P.DYNFEKVNRASMACGPMVKWAIAQIEYADMLKRVEP
C.elegans_dynein1 P.DWEFDKVNRASVACGPMVKWARAQLLYSTMLHKVEP

 © The Author(s)

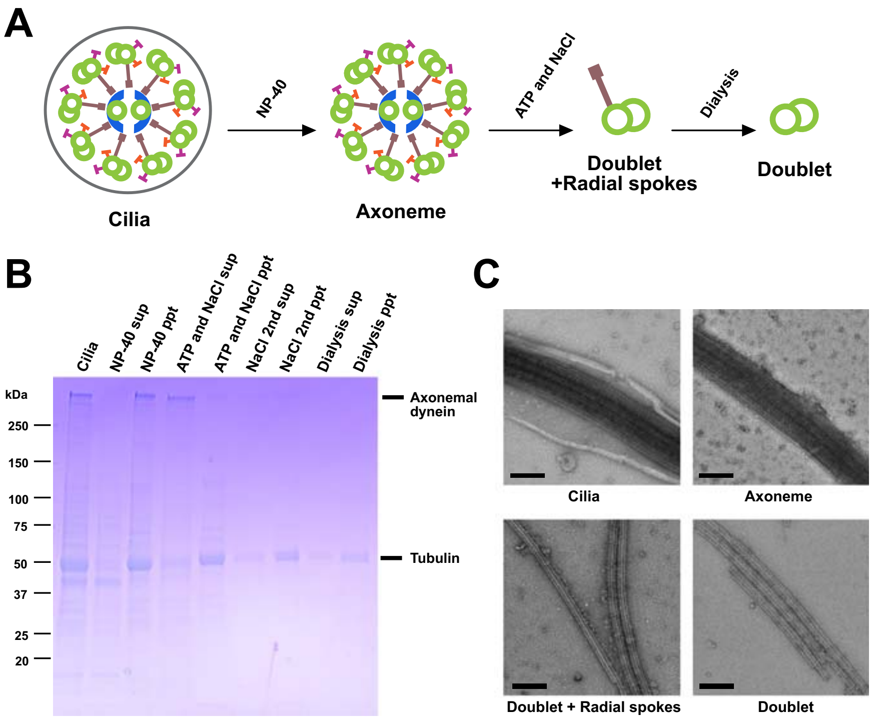

Figure EV2. Preparation of *Tetrahymena* doublets with clean outer surface.

(**A**) The workflow of doublet purification with a clean outer surface. (**B**) SDS-PAGE gel of sequential purification of *Tetrahymena* doublet. Associated proteins, especially axonemal dyneins, are removed while the tubulin band is visible. (**C**) Negative staining EM images of sequentially purified doublet samples. The doublets with clean outer surfaces were obtained. Scale bars: 200 nm.

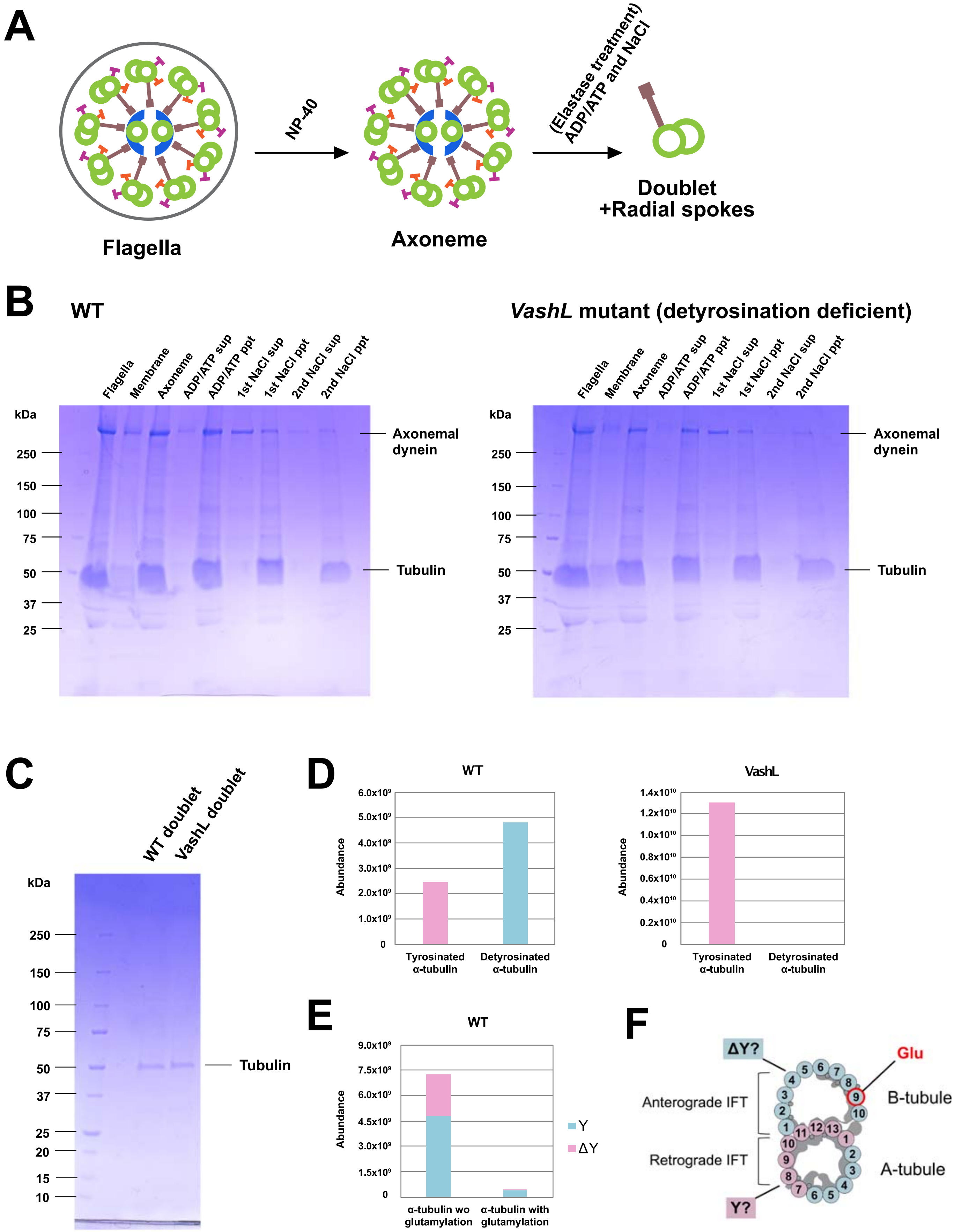
A
Flagella
NP-40
Axoneme
(Elastase treatment)
ADP/ATP and NaCl
Doublet
+Radial spokes
B
WT
VashL mutant (detyrosination deficient)
Flagella
Membrane
Axoneme
ADP/ATP sup
ADP/ATP ppt
1st NaCl sup
1st NaCl ppt
2nd NaCl sup
2nd NaCl ppt
kDa
250
150
100
75
50
37
25
Axonemal dynein
Tubulin
C
WT doublet
VashL doublet
kDa
250
150
100
75
50
37
25
20
15
10
Tubulin
D
WT
VashL
Abundance
Tyrosinated α-tubulin
Detyrosinated α-tubulin
E
WT
Abundance
α-tubulin wo glutamylation
α-tubulin with glutamylation
Y
ΔY
F
ΔY?
Y?
Glu
Anterograde IFT
Retrograde IFT
B-tubule
A-tubule

© The Author(s)

Figure EV3. Data related to *Chlamydomonas* doublets.

(**A**) The workflow of doublet purification from *Chlamydomonas* flagella. The elastase treatment was performed when doublets were used for EM analysis. For doublets used for the MS analysis, elastase treatment was not performed. (**B**) SDS-PAGE gels of sequential purification of *Chlamydomonas* doublets from either WT or *VashL* mutant cells. (**C**) SDS-PAGE gel to compare doublets purified from WT and *VashL Chlamydomonas* flagella. (**D**) Detection of tyrosinated and detyrosinated C-terminal peptides of α-tubulin by MS. Bar graphs show the abundance of tyrosinated and detyrosinated α-tubulin C-terminal peptides detected in WT (left) and *VashL* (right) doublet samples. In the WT sample, abundance values of tyrosinated peptides was 2.45×10^9 and that of detyrosinated peptides was 4.81×10^9. In contrast, only tyrosinated α-tubulin peptides were detected in the *VashL* mutant sample with abundance value of 1.30×10^9. (**E**) Detection of C-terminal peptides of α-tubulin with and without glutamylation by MS. The MS result of WT was re-analyzed based on whether C-terminal peptides have glutamylation, and the abundance of each form was plotted. The abundance values were as follows: ΔY wo E, 4.81×10^9; Y wo E, 2.45×10^9; ΔY with E, 3.83×10^8; Y with E, 1.10×10^8. (**F**) Schematic of possible localization of tyrosinated and detyrosinated PFs. Retrograde and anterograde IFT tracks are indicated. The location of glutamylated PF is based on (Alvarez Viar et al, 2024).

© The Author(s)

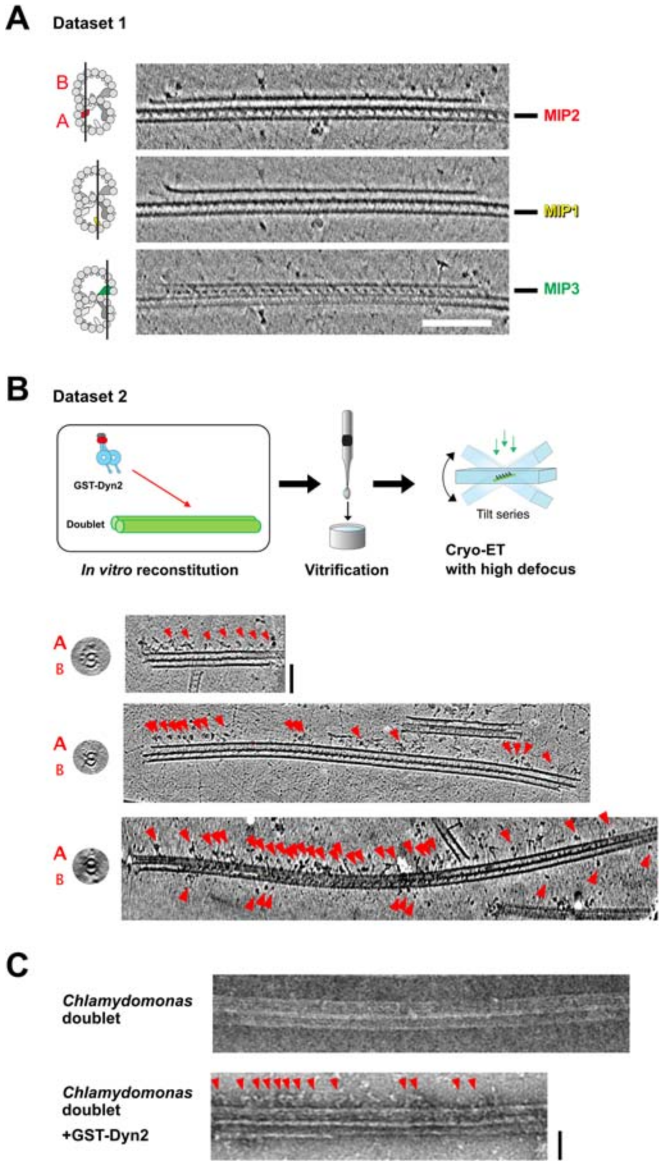

Figure EV4. Data related to decoration of doublet by GST-Dyn2.

(**A**) Longitudinal tomographic slice of the representative reconstructed doublet's 3D structure. Black lines in the illustrations of the doublet on the left indicate the sections on the right panels. MIP structures distinctive for A- or B-tubules are highlighted in the model and indicated in the tomographic slices. Naming and colorings are adopted from (Ichikawa et al, 2017; Ichikawa et al, 2019). Scale bar, 100 nm. (**B**) Cryo-ET workflow for dataset 2 (top), and representative tomographic slices (bottom). Cross-sectional (left) and longitudinal (right) views of the doublets decorated with GST-Dyn2 molecules (red arrowheads). A- and B-tubules of the doublets are indicated, with the A-tubule oriented toward the top. More GST-Dyn2 molecules were observed on the A-tubule sides. Tilt series for this dataset were acquired with a high defocus (−8 μm) instead of using the VPP. Scale bar, 100 nm. (**C**) Results of decoration of *Chlamydomonas* doublets by GST-Dyn2 molecules. Typical negative stain EM images of salt-treated *Chlamydomonas* doublet without (top) and with (bottom) incubation with GST-Dyn2. GST-Dyn2 molecules (red arrowheads) were observed to accumulate along one side of the doublets, presumably corresponding to the A-tubule side, judged by the thickness of the tubules. Scale bar, 50 nm.

 © The Author(s)

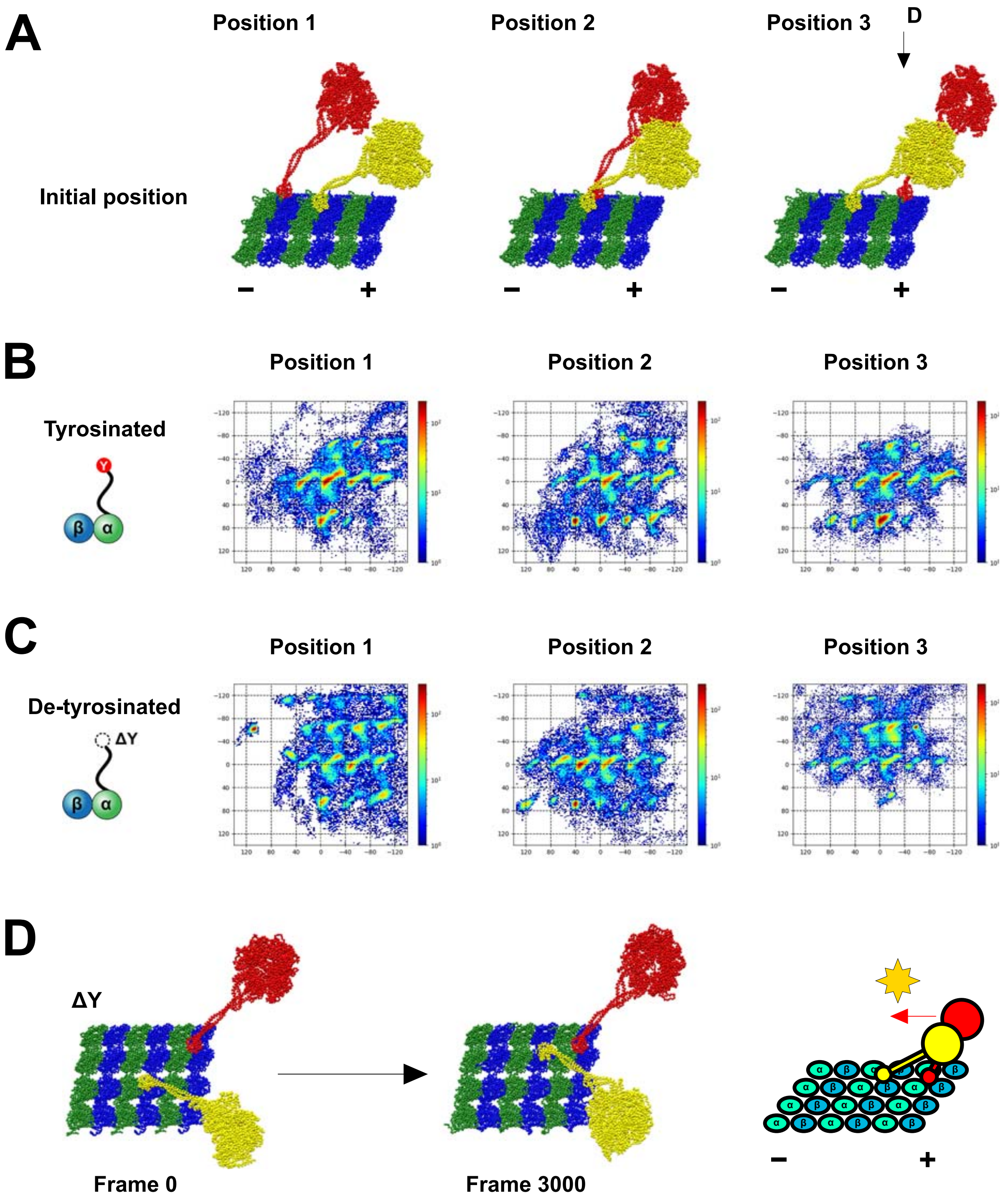

Figure EV5. Data related to MD simulations of dynein2 dimer.

(**A**) Initial structures of the MD simulations. Low-affinity dynein-2 motor domain structure (yellow) was placed at the center of the tubulin lattice with high-affinity dynein-2 (red) on the neighboring PF located at forward (position 1), adjacent (position 2), and backward (position 3). (−) and (+) indicate MT polarities. (**B, C**) Heatmaps of the positions of the MTBD of low-affinity dynein-2 on tubulin lattice with tyrosinated tubulins (**B**) and detyrosinated tubulins (**C**). 20 trajectories are overlaid and colored depending on the frequencies. Blue color corresponds to low frequency and red color represents higher frequency. (**D**) Snapshots of MD simulation results showing that the low-affinity leading head is blocking the high-affinity trailing head of dynein-2 in the detyrosinated tubulin lattice. The simulation was started from the initial state (frame 0, initial position 3 in (**A**)). In the simulated result (frame 3000), the low-affinity leading head moves in the direction of movement of the high-affinity trailing head. In the next cycle of the trailing head, the low-affinity leading head hinders the diffusional motion toward the minus end, and thereby, the dynein-2 dimer dissociates from the tubulin lattice (right panel). The view in (**D**) is indicated in (**A**).

© The Author(s)
